# The focus clinical research in intrahepatic cholangiocarcinoma

**DOI:** 10.1186/s40001-022-00741-9

**Published:** 2022-07-11

**Authors:** Yinghui Song, Mengting Cai, Yuhang Li, Sulai Liu

**Affiliations:** 1 Department of Nuclear Medicine, Hunan Provincial People’s Hospital, The First Affiliated Hospital of Hunan Normal University, Changsha, 410005 Hunan China; 2 Department of Hepatobiliary Surgery, Hunan Provincial People’s Hospital, The First Affiliated Hospital of Hunan Normal University Changsha, Changsha, 410005 Hunan People’s Republic of China; 3 Central Laboratory of The First, Affiliated Hospital of Hunan Normal University, Changsha, 410015 China

**Keywords:** Intrahepatic cholangiocarcinoma, Treatment, Clinical research

## Abstract

Intrahepatic cholangiocarcinoma (ICC), highly invasive and highly heterogeneous, has a poor prognosis. It has been confirmed that many risk factors are associated with ICC including intrahepatic lithiasis, primary sclerosing cholangitis (PSC), congenital abnormalities of the bile ducts, parasite infection, toxic exposures chronic liver disease (viral infection and cirrhosis) and metabolic abnormalities. In recent years, significant progress has been made in the clinical diagnosis and treatment of ICC. Advances in functional and molecular imaging techniques offer the possibility for more accurate preoperative assessment and detection of recurrence. Moreover, the combination of molecular typing and traditional clinical pathological typing provides accurate guarantee for clinical decision-making. Surgical resection is still the only radical treatment for ICC, while R0 resection, lymph node dissection, postoperative adjuvant therapy and recurrence resectomy have been confirmed to be beneficial for patients. New therapies including local therapy, molecular targeted therapy and immunotherapy are developing rapidly, which brings hopeful future for advanced ICC. The combination of traditional therapy and new therapy is the future development direction.

## Introduction

Intrahepatic cholangiocarcinoma (ICC), one of biliary tract cancers (BTC), originates from bile ducts of secondary or higher branches in the liver. It is anatomically different from other BTCs including gallbladder cancer (GBC), perihilar cholangiocarcinoma (PCCA) and distal cholangiocarcinoma (DCCA) [1]; while, ICC is also the second largest primary liver cancer in the worldwide [2].

There are various risk factors for ICC, the strongest being cysts and stones in the bile ducts, cirrhosis, and hepatitis B and C viruses. Some risk factors such as diabetes, albeit weakly, are increasing globally and may contribute to an increase in the incidence of CCA [3–7].

The incidence of ICC has increased significantly in recent years worldwide [8]. It is extremely challenging for the diagnosis and treatment of ICC for its insidious onset, strong heterogeneity, strong invasiveness, and poor prognosis. over the decades, great progress has been made in the diagnosis and treatment of ICC, which promote the update of guidelines and consensus [9, 10]. This article reviews the current status of clinical research on ICC.

### Accurate diagnosis and classification of ICC

The early detection rate of ICC is relatively low. First diagnosis of ICC is generally in late stage because of the insidious onset and the lack of sensitive detection and diagnostic markers. The traditional indicators of CA19-9 and carcinoembryonic antigen (CEA) have a low positive rate in early stage of ICC, which is difficult to detect ICC at early stage. Several advanced techniques such as liquid biopsy (blood [11] and bile [12]) and intestinal flora [13] for early diagnosis of biliary tract tumors, but the results still need further verification.

Then, molecular typing is beneficial to clinical decision-making of ICC. In addition to traditional clinicopathological typing based on growth pattern and histopathological features, molecular typing based on gene expression profiles [14], key gene mutation [15] or multi-omics techniques [16] has been proposed to guide clinical therapeutic decisions in recent years. Combining molecular typing with traditional clinicopathological typing, and converting the complicated typing in laboratory into practical clinical typing detection, which will be the focus of attention in the future.

Imaging methods are still the main means for diagnosis, preoperative assessment and recurrence monitoring of ICC. The liver contrast-enhanced ultrasound, enhanced CT and MRI are commonly used in diagnosis of ICC [17–19]. MRI can identify the local and periductal invasion of tumor accurately, display the bile duct system clearly and completely, especially suitable for the evaluation of periductal infiltrative ICC [20]. Thus, MRI can accurately identify the *T* stage of ICC, while PET–CT is used in preoperative evaluation for detecting lymph node metastasis (*N* stage), distant metastasis (*M* stage) and even occult metastatic disease, as well as detection of the site of disease recurrence with high sensitivity and specificity [21, 22]. The combination of the two imaging methods can identify tumor stage comprehensively and provide precise information for resectable tumor. Molecular imaging technologies including PET–MR [23, 24] and fluorescence imaging [25, 26] are expected to be vital roles in clinical diagnosis and decision-making of ICC in recent years.

The clinicopathological staging systems including American Joint Council on Cancer (AJCC) staging [27], Japanese Society of Hepato-Biliary-Pancreatic Surgery (JSHBPS) staging [28] and Liver Cancer Study Group Japan (LCSGJ) staging [29] are still the main tool for clinical decision-making and prognostic assessment of ICC. AJCC staging, based on tumor size, number, invasion of large vessels, direct invasion of adjacent tissues, lymph node metastasis and distant metastasis, has been widely used in various guidelines for clinical prognostic assessment and therapeutic decision-making of ICC.

### Surgical treatment of ICC

Radical surgical resection remains the only cure treatment for ICC. The postoperative survival rate of patients with ICC is gradually improved (5-year survival rate is 30–40%) [30]. An aggressive surgical approach including extensive liver resection plus extended systematic lymph node dissection is currently recommended to improve prognosis [31]. But the incidence of local recurrence and distant metastasis is still high (60–70%), which seriously affects the long-term postoperative survival of patients [32]. The surgical margin, laparoscopic liver resection (LLR), routine combined lymph node dissection, associating liver partition and portal vein ligation for staged hepatectomy (ALPPS), secondary liver resection after recurrence and prognostic prediction model are the research hotspots.

As well known, high invasiveness is an important biological feature of ICC. The pros and cons of surgical resection including anatomic resection and non-anatomic resection are still inconclusive. Although some studies have revealed that anatomic hepatectomy can reduce postoperative recurrence rate and improve overall prognosis of ICC patients [33], especially for ICC patients with TNM stage I tumors > 5 cm and TNM stage II without vascular invasion. However, prospective randomized controlled studies are needed to clarify this controversial point. Surgical margin is a vital factor affecting postoperative recurrence and survival. Therefore, many guidelines stipulate that ICC surgery should follow with the principle of R0 resection [18, 34], but the definition of the width of R0 resection is still inconclusive yet. In recent years, researchers have found that surgical margins with width > 1 cm have better survival benefits after surgery [35, 36]. Thus, an increasing evidence support to make the surgical margin wide as possible while ensuring there is enough remaining liver volume [37].

In recent years, the laparoscopic technique has developed rapidly, providing a new surgical option for ICC patients. Researchers have shown that LLR for ICC was associated with fewer blood transfusion, higher R0 resection, shorter length of stay (LOS), less overall morbidities, and less death due to tumor recurrence compared with open liver resection (OLR) [36]. There were no significant differences in surgical duration, blood loss, LN metastasis, major morbidities, mortality, tumor recurrence, 3 year OS and disease-free survival (DFS), and 5 year DFS [36]. However, the current results are all from retrospective studies, which means that prospective randomized controlled studies are still needed to provide high-level evidence in the future. On the other hand, it has become increasingly strict for regional lymph node dissection in recent years, which has also put forward higher technical requirements for LLR. However, the effect of LLR on the quality of regional lymph node dissection remains controversial from the results of current retrospective studies [38, 39].

Lymph node metastasis is an important biological feature of ICC, and it is also the most common metastasis pathway of ICC, which is significantly associated with poor prognosis. The results of lymph node dissection can be used to guide staging and determine indications for adjuvant chemotherapy. It has long been controversial whether lymph node dissection should be performed routinely in ICC. Recent updated guidelines and expert consensus recommend routine intraoperative combined with regional lymph node dissection including hepatoduodenal ligament, perihepatic artery and head of pancreas, and the number of detected lymph nodes should not be less than 6 [40]. At present, the acceptance of lymph node dissection in western countries is significantly higher than that in eastern countries [41]. However, it remains controversial whether preventive negative lymph node dissection can improve the prognosis of ICC patients [42]. The median OS after radical resection is about 30 months, and the 5-year overall survival rate is generally up to 40%, and patients with negative margins (R0 resection) and no lymph node involvement can achieve better survival. The 5-year survival rate of them can be as high as 63% [43]. Most of the previous studies were single-center retrospective studies, lack of uniformity in the scope of lymph node dissection and there may be a selection bias in it, which make rigorous prospective studies needed. In addition, it is also the direction of future research to find people who really benefit from lymph node dissection for individualized treatment [44].

Associating liver partition and portal vein ligation for staged hepatectomy (ALPPS) may provide a chance of radical resection for some patients with locally advanced ICC. Previous studies have shown that the incidence of perioperative complications and mortality of ALPPS for ICC patients are higher than those for patients with colorectal cancer and liver metastasis [45]. But the surgical safety has been significantly improved in recent years. Compared with chemotherapy alone, the patients with locally advanced ICC have better prognosis by ALPPS [46].

The postoperative recurrence rate of ICC is 60–70%, and the most common is intrahepatic recurrence. Only about 9% of recurrent patients have received hepatectomy again at present [47]. Recent studies have revealed that secondary liver resection after recurrence can significantly prolong the overall survival (OS) of patients and some patients still have the possibility of long-term survival or even cure compared with palliative treatment [48]. It has been reported that the prognosis of patients receiving secondary hepatectomy for ICC recurrence is similar to that of patients receiving primary hepatectomy [32]. Therefore, current guidelines recommend re-surgical resection to further improve patient prognosis for resectable recurrent intrahepatic lesions [18].

The prognostic prediction model for ICC after surgical resection mainly includes: (1) clinical staging system such as AJCC staging [27], Liver Cancer Study Group of Japan (LCSGJ) staging [29], etc. (2) Scoring systems such as FUDAN scoring (the necessary clinical parameters can be easily obtained preoperatively through imaging and serum biochemical tests) [49], MEGNA scoring (easy to accurate estimation of patient survival after hepatectomy) [50], etc. (3) New prediction models such as nomogram, et al. [51]. Clinical staging systems are practical, but less accurate. Although nomogram has high predictive efficiency, it has cumbersome operation and poor clinical practicability, while the scoring system has a relatively simple operation and certain practicability. On the other hand, traditional clinical prediction models mainly incorporate clinicopathological features. Tumor genomic stratification should combine clinicopathological features to provide more accurate information for prognosis prediction of ICC in the future.

### Liver transplantation for ICC

Earlier studies found that patients with ICC had a poor prognosis after liver transplantation, and most centers regarded ICC as contraindicated for liver transplantation, which has changed in recent years [52, 53]. Based on two multi-center retrospective studies, Sapisochin et al. proposed that “extremely early ICC” (single tumor < 2 cm in diameter) with liver cirrhosis achieved a better survival through liver transplantation, the 1-, 3-, and 5-year survival rates were 93%, 84%, and 65%, respectively [54]. This viewpoint is still controversial and lacks support from high-quality clinical evidence. It is believed that the combined treatment of neoadjuvant chemoradiotherapy and liver transplantation may be an effective treatment mode for locally advanced ICC [55], but all the evidence come from small sample studies. Therefore, in the International Liver Transplantation Society (ILTS) in 2020, it is still not recommended to diagnose early or locally advanced ICC through liver transplantation therapy outside clinical trials in the absence of high-quality evidence-based medical evidence [56].

### Local treatment of unresectable ICC

Intra-arterial therapies (IAT) include transcatheter arterial chemoembolization (TACE), trans-arterial radioembolization (TARE) and hepatic artery infusion (HAI). Scheuermann U et al. have confirmed that TACE can achieve a similar effect to palliative surgical resection in unresectable locally advanced ICC [57], while postoperative adjuvant TACE can significantly reduce tumor recurrence and improve survival [58]. A recent study of postoperative adjuvant TACE for hepatitis B-related intrahepatic cholangiocarcinoma included 9 patients who received postoperative adjuvant TACE, and 33 patients who underwent surgery only. There were significant differences in the 1-, 3-, and 5-year survival rates between the TACE group and surgery group, 88.9%, 77.8%, 66.7% vs. 63.6%, 30.8%, 13%, respectively (*P* = 0.037) [59]. At the same time, multivariate analysis also confirmed that postoperative adjuvant TACE can improve the long-term survival of patients after surgery (HR: 0. 123, 95% confidence interval 0. 023–0. 643, *P* = 0. 013). In a previous clinical study [60], 53 patients were treated with percutaneous thermal ablation with simultaneous TACE, the RR was 80.7%, and the median PFS and median OS were 7.2 months and 20.9 months, respectively. The 1-, 2-, and 3-year cumulative survival rates were 72.6%, 39.1%, and 24.3%, respectively. The cumulative PFS rates at 6, 12, and 18 months were 58.3%, 40.4%, and 24.2%, respectively. This study showed that percutaneous thermal ablation with TACE was safe and effective in the treatment of advanced ICC. TARE had the dual advantages of interventional embolization and local radiotherapy by infusion of yttrium 90, and it has shown positive therapeutic significance in locally advanced ICC and recurrent ICC. Through a recent phase II study, researchers also revealed that TARE combined with first-line chemotherapy had a disease control rate of 98% and a median survival time of 22 months in the treatment of locally advanced ICC, which was higher than current single first-line chemotherapy regimens [61]. At present, a phase III trial is underway. On the other hand, HAI has shown some efficacy in locally advanced ICC. The latest phase II study illustrated that the median survival of HAI combined with first-line chemotherapy in ICC was 24 months [62]. Meanwhile, HAI or TARE combined with chemotherapy made some initially unresectable patients into surgical intervention in the above phase II studies, which provided a possibility for translational treatment of locally advanced ICC [61, 62]. Overall, the improved trans-arterial interventional therapy has become a new treatment method for unresectable ICC.

The commonly used radiotherapy methods are external radiation therapy such as stereotactic body radiation therapy (SBRT) and intensity modulated radiation therapy (IMRT), brachytherapy such as selective internal radiation therapy (SIRT), and new radiotherapy techniques developed in recent years such as proton therapy. Hong TS et al. have shown that external beam radiation therapy or high-dose low-fraction proton therapy combined with concurrent chemotherapy can achieve a better local control rate and OS rate for locally advanced ICC, and relieve symptoms inclusive of cancer pain in ICC patients [63], and can be a valuable clinical treatment option [16]. However, it remains controversial that the benefits of external beam radiation therapy or new radiotherapy combined with concurrent chemotherapy for the clinical treatment of advanced ICC [64]. In a prospective and retrospective study, SBRT resulted in local control rates ranging from 65 to 100% in selected patients, with a median OS of 11 to 35.5 months (median 15 months) [64].

Radiofrequency ablation (RFA) can be used as an alternative treatment for patients with small single ICC that cannot tolerate surgery, and its effect is significantly better than systematic chemotherapy [65]. A meta-analysis showed [66] that patients treated with RFA before biliary stent implantation had a longer stent obstruction time of 13 days and a longer survival time of 37 days, respectively, compared with patients who did not receive RFA, highlighting the advantages of RFA in the comprehensive treatment of malignant biliary obstruction. For unresectable or recurrent ICC, it can also bring survival benefits by RFA combined with chemotherapy, but further clinical studies are still needed. Photodynamic therapy (PDT) is also an ablation therapy with the advantages of wide applicability, less adverse reactions, less injury, simplicity and ease of operation, and low postoperative recurrence rate. Wentrup et al. and ParR et al. compared the efficacy of PDT combined with different drugs and PDT alone. Both studies proved that PDT combined with chemotherapy can enhance the efficacy, prolong patient survival, and reduce mortality. It can be seen that the application of PDT in patients with unresectable extrahepatic cholangiocarcinoma can relieve clinical symptoms, reduce tumor volume, delay tumor growth, improve quality of life, and prolong patient survival [67, 68]. Therefore, RFA offers more options for ICC treatment.

### Systemic therapy for ICC

In retrospective studies, it had been proved that postoperative adjuvant chemotherapy has a potential survival benefit for ICC patients, especially for high-risk patients with positive lymph node or R1 resection, but there is currently lack of specific chemotherapy regimens. In bile duct cancer adjuvant trial (BCAT) and PRODIGE 12-ACCORD 18-UNICANCER GI study, it had been demonstrated that adjuvant chemotherapy regimen based on gemcitabine showed no survival benefit compared with observation group. While in resected biliary tract cancer (BILCAP), it had been illustrated that capecitabine could significantly prolong OS and disease-free survival (DFS) [69–71]. Therefore, 6 months of capecitabine adjuvant therapy is recommended for ICC patients undergoing radical resection at guidelines for adjuvant treatment of biliary tumors in the American Society of Clinical Oncology (ASCO). There is also cumulative evidence that adjuvant TACE or radiotherapy after surgery of ICC can significantly reduce the risk of tumor recurrence and bring survival benefits for high-risk patients in other studies [58, 72], while postoperative antiviral therapy for hepatitis B-related ICC can significantly reduce tumor recurrence [73]. Thus, postoperative adjuvant therapy can significantly improve the survival of ICC especially for the high-risk patients.

Gemcitabine combined with platinum-based chemotherapy regimen is still a first-line option for locally advanced ICC and metastatic ICC, and new first-line chemotherapy options are being explored. In head-to-head phase III trials, GS (gemcitabine and cisplatin) and XELOX (capecitabine and oxaliplatin) had similar survival benefits to current first-line regimens, while GCS(gemcitabine, cisplatin and S-1) was better than GC (gemcitabine and cisplatin) [70, 74, 75]. Moreover, GCS has better tolerability and convenience, while the combination of albumin-bound paclitaxel and conventional chemotherapy also achieved preliminary effect on patients with advanced ICC, and median PFS and OS reached similar or even better levels than the current first-line regimen [76, 77]. Modified FOLFIRINOX had promising efficacy and favorable tolerance in patients with advanced ICC [78]. Phase III clinical trials of modified FOLFIRINOX is currently ongoing, which is expected to provide more first-line chemotherapy options for advanced ICC.

There is no standard protocol for second-line treatment of ICC. ABC-06, the first phase III trial to explore second-line chemotherapy for advanced ICC, had demonstrated that mFOLFOX combined with best supportive therapy could improve OS of ICC patients significantly. This finding supported that mFOLFOX could be used as the second-line standard for advanced ICC [79].

The value of adjuvant therapy after R0 resection for patients with intrahepatic and perihilar cholangiocarcinoma has been ongoing. For some inoperable patients, induction therapy may be considered first. The results from a cohort of 10 studies enrolled a total of 334 patients with locally advanced PCC and ICC who received induction therapy showed that 180 (53.9%) patients underwent resection after induction therapy, and 115 (63.9%) patients underwent R0 resection [80]. Pooled OS data showed that chemotherapy plus resection resulted in better OS than chemotherapy alone (HR = 0.31, 95% CI 0.19–0.50; *P* < 0.0001). In addition, the treatment was well tolerated with a low incidence of toxicity in the included studies. These findings may suggest that surgically selected patients after induction therapy with initially locally advanced or ICC are safe and feasible.

Molecular targets such as FGFR2 fusion, IDH1/2 mutation, HER2 amplification and NTRK fusion are expected to be used for the targeted treatment of ICC, but most of them are currently in the phase clinical research [81]. The incidence of FGFR2 gene fusion in ICC is 10% to 20%. According to the result of a phase II clinical trial, the objective response rate of FGFR-targeted treatment for advanced ICC with previous treatment failure was 20.7–35.5%, the disease control rate was 79–83.6%, and the median survival time was 21.1 months [81]. Based on the FIGHT-202 trial, the US Food and Drug Administration (FDA) has recently approved pemigatinib for previously treated ICC with FGFR2 fusions or rearrangements [82]. Meanwhile, a phase III clinical trial comparing FGFR inhibitors with first-line chemotherapy for ICC patients with FGFR2 fusions is ongoing, which is expected to revolutionize the clinical treatment of ICC patients with FGFR2 fusions. In addition, there are other potentially targeted low-frequency mutations in ICC, such as BRAF V600E mutation, HER2 amplification and NTRK fusion, etc. Although clinical trials are difficult to conduct due to low incidence, basket trials have confirmed the therapeutic value of these targets in ICC [81].

PD-1/PD-L1 inhibitors have been found to be effective against malignant tumors such as gastric cancer and liver cancer [83]. There are many other different pembrolizumab combination regimens in clinical studies on advanced cholangiocarcinoma. It was reported that pembrolizumab combined with granulocyte-macrophage colony-stimulating factor in the treatment of advanced biliary tract tumors, with 27 cases from a phase II clinical trial. Five of the patients (74% ICC) (19%, 1 microsatellite unstable, 4 microsatellites stable) achieved partial responses, with a 6-month disease-free survival rate of 35%. Thirty percent of cases were PD-L1 positive (≥ 1%), but were not associated with objective response rates or 6-month disease-free survival. Targeted PD-1 therapy has been approved for advanced ICC with mismatch repair deficiency (dMMR) or microsatellite instability high (MSI-H). The efficacy of immune checkpoint therapy in pMMR/MSS TYPE ICC is unclear, and the prognostic effect of TMB and PDL-1 expression status has not been concluded [84–86]. In future clinical studies, the combination of immune checkpoint therapy with chemotherapy, targeted therapy and local therapy will be a focus of attention. In addition, M7824, a new generation of bifunctional immune-drug targeting both PD-L1 and TGF-β, further improves the efficacy of immunotherapy by effectively inhibiting immune escape. According to clinical data presented at the 2018 European Society of Internal Oncology (ESMO) congress, M7824 has an overall response rate (ORR) of 20% in patients with ICC who have failed first-line treatment and is not affected by PD-L1 level (response duration of 8.3–13.9 months), and has been granted orphan drug status by FDA for cholangiocarcinoma. Phase III trials of M7824 in combination with chemotherapy for first-line treatment of advanced ICC are also ongoing [87].

In addition to immune checkpoint therapy, tumor vaccine [88] and immune cell therapy [89] have been successfully used to treat advanced ICC. However, most of them are limited to small samples and case report at present, and there is still a lack of confirmation of large samples and high-quality prospective randomized controlled studies.

### Conclusion

The etiology of ICC is complex and unclear yet. The tumor is very aggressive and the prognosis is poor. In recent years, significant progress has been made in the clinical diagnosis and treatment of ICC. Firstly, advances in imaging detection methods have provided more accurate preoperative assessment and recurrence monitoring especially functional and molecular imaging. Secondly, the combination of molecular typing and traditional clinicopathological typing provides more accurate basis for the diagnosis and treatment of ICC. In addition, radical surgical resection remains the only curative treatment for ICC, and the importance of R0 resection, lymph node dissection, postoperative adjuvant therapy and recurrence resection has been confirmed by increasing evidence. The value of laparoscopic minimally invasive surgery, ALPPS and liver transplantation requires more high-level clinical research evidence. Last but not least, the rapid development of new therapies such as local therapy, targeted therapy and immunotherapy has brought new hope for the treatment of advanced ICC. It is inevitable in the development of ICC treatment including accurate molecular typing, sensitivity to targeted therapy and personalized therapy. The sequence and combination of traditional treatment and novel treatment will further improve the curative effect of ICC.

## Data Availability

Data and materials are included in the manuscript.
