# Correction to: The focus clinical research in intrahepatic cholangiocarcinoma

**DOI:** 10.1186/s40001-022-00791-z

**Published:** 2022-09-01

**Authors:** Yinghui Song, Mengting Cai, Yuhang Li, Sulai Liu

**Affiliations:** 1 Central Laboratory of The First Affiliated Hospital of Hunan Normal University, Changsha, 410015 China; 2 Department of Nuclear Medicine, Hunan Provincial People’s Hospital, The First Affiliated Hospital of Hunan Normal University, Changsha, 410015 China; 3 Department of Hepatobiliary Surgery, Hunan Provincial People’s Hospital, The First Affiliated Hospital of Hunan Normal University, Changsha, 410015 China

## Correction to: European Journal of Medical Research (2022) 27:116 10.1186/s40001-022-00741-9

In the original publication of the article [1] the affiliation details of the first author, Yinghui Song was incorrectly published. The corrected affiliation is given in this Correction. The original article has been corrected.
